# Altered Functional Interactions of Inhibition Regions in Cognitively Normal Parkinson’s Disease

**DOI:** 10.3389/fnagi.2018.00331

**Published:** 2018-10-23

**Authors:** Deborah L. Harrington, Qian Shen, Rebecca J. Theilmann, Gabriel N. Castillo, Irene Litvan, J. Vincent Filoteo, Mingxiong Huang, Roland R. Lee

**Affiliations:** ^1^Cognitive Neuroimaging Laboratory, Research Service, VA San Diego Healthcare System, San Diego, CA, United States; ^2^Department of Radiology, University of California, San Diego, La Jolla, CA, United States; ^3^Department of Neurosciences, University of California, San Diego, La Jolla, CA, United States; ^4^Psychology Service, VA San Diego Healthcare System, San Diego, CA, United States; ^5^Department of Psychiatry, University of California, San Diego, La Jolla, CA, United States; ^6^Department of Radiology, VA San Diego Healthcare System, San Diego, CA, United States

**Keywords:** Parkinson’s disease, response inhibition, cognition, task-activated functional MRI, context-dependent connectivity, diffusion tensor imaging, brain volume

## Abstract

Deficient inhibitory control in Parkinson’s disease (PD) is often observed in situations requiring inhibition of impulsive or prepotent behaviors. Although activation of the right-hemisphere frontal-basal ganglia response inhibition network is partly altered in PD, disturbances in interactions of these regions are poorly understood, especially in patients without cognitive impairment. The present study investigated context-dependent connectivity of response inhibition regions in PD patients with normal cognition and control participants who underwent fMRI while performing a stop signal task. PD participants were tested off antiparkinsonian medication. To determine if functional disturbances depended on underlying brain structure, aberrant connectivity was correlated with brain volume and white-matter tissue diffusivity. We found no group differences in response inhibition proficiency. Yet the PD group showed functional reorganization in the long-range connectivity of inhibition regions, despite preserved within network connectivity. Successful inhibition in PD differed from the controls by strengthened connectivity of cortical regions, namely the right dorsolateral prefrontal cortex, pre-supplementary motor area and right caudal inferior frontal gyrus, largely with ventral and dorsal attention regions, but also the substantia nigra and default mode network regions. Successful inhibition in controls was distinguished by strengthened connectivity of the right rostral inferior frontal gyrus and subcortical inhibition nodes (right caudate, substantia nigra, and subthalamic nucleus). In both groups, the strength of context-dependent connectivity correlated with various indices of response inhibition performance. Mechanisms that may underlie aberrantly stronger context-specific connectivity include reduced coherence within reorganized systems, compensatory mechanisms, and/or the reorganization of intrinsic networks. In PD, but not controls, abnormally strengthened connectivity was linked to individual differences in underlying brain volumes and tissue diffusivity, despite no group differences in structural variables. The pattern of structural-functional associations suggested that subtle decreases in tissue diffusivity of underlying tracts and posterior cortical volumes may undermine the enhancement of normal cortical-striatal connectivity or cause strengthening in cortical-cortical connectivity. These novel findings demonstrate that functionally reorganized interactions of inhibition regions predates the development of inhibition deficits and clinically significant cognitive impairment in PD. We speculate that altered interactions of inhibition regions with attention-related networks and the dopaminergic system may presage future decline in inhibitory control.

## Introduction

Executive dysfunction is the most frequently reported cognitive disability in Parkinson’s disease (PD). A feature of executive dysfunction is diminished response inhibition, which affects the selection of behaviors and decisions about when or whether to act. Response inhibition deficits in PD are found on a variety of tasks (e.g., Stroop, Go No-Go, Stop Signal) requiring the inhibition of impulsive or prepotent responses (Obeso et al., 2011a; Nombela et al., 2014; Cerasa et al., 2015; Manza et al., 2017), and are of particular importance owing to their association with freezing of gait (Vandenbossche et al., 2012; Fling et al., 2013; Cohen et al., 2014; Walton et al., 2015), a disabling manifestation of the disease in some patients.

Our current understanding of the brain mechanisms that support response inhibition disturbances in PD is incomplete. Failure to inhibit impulsive behaviors is linked to hyperactivity of the subthalamic nucleus (StN) (Alegre et al., 2013; Schmidt et al., 2013). The StN is a key node of a cortical-subcortical right-hemisphere inhibition network, which is also comprised of the inferior frontal gyrus (IFG), pre-supplementary motor area (preSMA), primary motor cortex, and basal ganglia [caudate nucleus, substantia nigra (SN)] (Chambers et al., 2009; Wiecki and Frank, 2013; Aron et al., 2014; Morein-Zamir and Robbins, 2015). This network has been widely studied using the stop signal task (SST), which assesses the ability to successfully inhibit a response that is already started to a prepotent Go stimulus. Successful canceling of an action while performing the SST during functional MRI (fMRI) is associated with decreased activation in PD patients on antiparkinsonian medication (PD ON) relative to controls in several regions of the inhibition network (right IFG, preSMA, bilateral caudate/putamen, StN) (Rae et al., 2016). Hypoactivation of the right IFG is most consistently reported in SST studies of PD ON (Ye et al., 2014, 2015, 2016) and *de novo* PD patients (Vriend et al., 2015), suggesting that decreased activation is unrelated to chronic medication effects.

Notably, much of what we know about changes in the response inhibition network in PD comes from analyses of regional activation, which are insensitive to disturbances in the interactions of inhibition nodes amongst themselves or with other brain regions. Recently, functional connectivity between the right IFG and the striatum during successful inhibition (relative to Go trials) was reported to be weaker in PD ON patients than in controls (Ye et al., 2015), but connectivity with other key inhibition nodes was not studied. In addition, dynamic causal modeling failed to characterize functional interactions between four inhibition network nodes (preSMA, IFG, StN, motor cortex) in PD ON patients, as it did in controls (Rae et al., 2016). This negative result may relate to heterogeneity among patients and/or the omission of nodes from the models that are important in inhibitory control in PD. In this regard, interactions of the inhibition network with other centers, in addition to the IFG, might also explain difficulties in stopping actions. Indeed, the inhibition network is supported by a central hub of the salience network, the anterior insula (Hampshire and Sharp, 2015), and an executive processing center, the dorsolateral prefrontal cortex (DLPFC) (Jahanshahi et al., 2015b; Morein-Zamir and Robbins, 2015; Zhang et al., 2017), both of which can be dysfunction in PD.

The present study builds upon previous research by characterizing disease-related disturbances in the context-dependent connectivity of regions associated with response inhibition processes. Response inhibition is thought to involve a right-hemisphere biased frontal-striatal-subthalamic network (Jahanshahi et al., 2015b). However, the specific regions of the network and whether regions play a direct or supportive role in inhibition remain debated (Aron, 2007; Chamberlain et al., 2009; Hampshire, 2015; Hampshire and Sharp, 2015; Limongi and Perez, 2017; Bartoli et al., 2018; Hung et al., 2018). For this reason, we focused on regions commonly implicated in response inhibition (Aron, 2007; Hampshire and Sharp, 2015; Hung et al., 2018), regardless of their purported roles, as altered functioning in any of these regions could adversely affect inhibitory control in PD. Regions of interest included the right IFG, preSMA, anterior insula, DLPFC, caudate nucleus, StN, and SN. Unlike past studies, a cognitively normal PD cohort (Litvan et al., 2012) was studied to control for the potential effects of mild cognitive impairment (MCI) on response inhibition proficiency. Healthy control and PD OFF participants underwent fMRI while performing the SST. An advantage of the SST is that it allows for an analysis of networks associated with successful inhibition (Stop correct > Go correct) and performance accuracy (Stop correct > Stop incorrect), which differ in their engagement of some brain networks (Zhang and Li, 2012). We predicted that PD patients would exhibit abnormal context-dependent connectivity of the response inhibition nodes, including centers known to support higher-level executive functions (DLPFC). Because structural changes in the brain may affect functional changes, context-specific connectivity disturbances were correlated with brain volumes and white-matter diffusivity in underlying tracts.

## Materials and Methods

### Participants

The Institutional Review Board at the VA San Diego Healthcare System approved the study. All subjects provided signed written informed consent. The sample consisted of 28 PD participants who met the PD United Kingdom Brain Bank Criteria and 29 healthy controls. Exclusion criteria included metal in the head, neurological diagnoses other than PD, psychiatric diagnoses, history of alcohol or substance abuse, positive MRI findings (e.g., infarcts, vascular disease), use of anticholinergics or cognitive medications (e.g., Donepezil), and complaints of cognitive deficits. PD volunteers with axial tremors or upper/lower limb tremors that might cause head motion were excluded. Volunteers were excluded if they met the Movement Disorders Society Level II criteria for PD-MCI (Litvan et al., 2012), hereafter referred to as Level II criteria. Using two tests for each of five domains (Table 1), MCI was defined as >1.5 standard deviations below the control group mean on at least two tests in a single domain or different domains (Goldman et al., 2018). PD volunteers were also excluded if they reported problems with cognitive functioning in daily life [Unified Parkinson’s Disease Rating Scale (UPDRS) Part I, item 1]. Neuropsychological testing was conducted when patients were taking antiparkinsonian medication. For MRI scanning, patients stopped medication overnight for a minimum of 14 h.

**Table 1:** Demographic, clinical and cognitive variables.

	Parkinson’s	Control	*p*^a^	$\eta_{p}^{2}$
Age (years)	67.3 (7.6)	68.3 (7.2)	0.89	0.00
Education (years)	17.1 (2.3)	16.7 (1.7)	0.99	0.00
Sex (% females)	29.0	62.0	0.01	
Handedness (% right handed)	89.3	89.7	0.55	
Wechsler Test of Adult Reading	44.9 (4.4)	45.3 (4.1)	0.52	0.01
Mini-Mental Status Exam	29.3 (0.9)	29.5 (0.7)	0.42	0.01
Hamilton Depression Scale	3.6 (2.3)	2.1 (2.7)	0.06	0.07
Epworth Sleepiness Scale	8.8 (4.1)	7.2 (2.6)	0.13	0.05
Disease duration (years)	5.4 (3.9)			
Levodopa dosage equivalence^b^	735.2 (414.3)			
UPDRS Total Motor ON^c^	26.9 (12.4)			
Tremor ON	2.5 (2.0)			
PIGD ON	2.2 (1.9)			
UPDRS Total Motor OFF	36.7 (13.7)			
Tremor OFF	3.4 (2.3)			
PIGD OFF	2.6 (1.9)			
**Attention and Working Memory**				
Adaptive Digit Ordering (maximal span)	5.5 (1.1)	5.6 (1.3)	0.74	0.00
Attention subscale (MDRS)	36.1 (1.2)	36.2 (1.0)	0.98	0.00
**Executive**				
Verbal Fluency-Letters (DKEFS)	38.9 (11.4)	46.2 (13.5)	0.02	0.09
Inhibition/Switching (DKEFS)	68.4 (19.9)	63.1 (16.9)	0.38	0.02
**Memory**				
CVLT-2 long delay free recall	9.4 (3.5)	11.5 (2.9)	0.06	0.06
Logical Memory II (WMS-III)	29.5 (5.5)	31.5 (8.6)	0.47	0.01
**Visuospatial**				
Judgment of Line Orientation	24.5 (4.6)	24.9 (3.3)	0.42	0.01
Hooper Visual Organization	25.5 (2.3)	25.8 (2.9)	0.40	0.01
**Language**				
Boston Naming	57.8 (2.1)	57.4 (2.3)	0.62	0.01
Similarities (WAIS-IV)	28.6 (4.0)	28.4 (5.4)	0.86	0.00


*CVLT-2, California Verbal Learning Test version 2; DKEFS, Delis Kaplan Executive Function System; MDRS, Mattis Dementia Rating Scale; PIGD, Postural instability/gait difficulty on the UPDRS; WAIS IV, Wechsler Adult Intelligence Scale III; WMS III, Wechsler Memory Scale III; UPDRS, Unified Parkinson’s Disease Rating Scale. ^*a*^*F* and chi-square (sex, handedness) statistics. All *F* tests used ANCOVA, adjusting for sex. Tabled values are unadjusted raw score means (standard deviations). ^*b*^Levodopa dosage equivalence was calculated using the method of Tomlinson et al., 2010. ^*c*^Total motor, tremor, and PIGD scores were significantly greater OFF than ON medications (*F* = 99.5, *p* < 0.00001; *F* = 14.1, *p* < 0.001; *F* = 7.1, *p* < 0.013, respectively). Tremor and postural instability/gait (PIGD) scores were computed based on Jankovic et al. (1990).*

The groups did not differ in age, educational level, or premorbid intelligence (Wechsler Test of Adult Reading), but the control group had a greater percentage of females (Table 1). PD participants were taking dopamine agonist monotherapy (*n* = 2), levodopa monotherapy (*n* = 5), or levodopa combination therapy (*n* = 21), and were in Hoehn and Yahr stages 1 (11%), 2 (53%) and 3 (36%). UPDRS total motor, tremor, and postural instability/gait disorder (PIGD) symptoms (Jankovic et al., 1990) were significantly greater off than on medication (Table 1).

### Stop Signal Task

The SST consisted of a series of Go (75%) and Stop (25%) reaction-time trials that were presented in a pseudorandom order (i.e., 1 Stop trial for every 3 Go trials) (Aron and Poldrack, 2006). Each trial began with a 500 ms warning signal consisting of a central fixation cross. This was followed by a green triangle that was presented to the left or right of the fixation cross. On Go trials, the participant responded as quickly as possible, making either a left or right key press using the index and middle fingers of the right hand. On Stop trials, the green triangle turned red after a variable step-up step-down delay, signaling the participant to attempt to stop his/her response. The stop signal duration (SSD) is the time between the Go signal (green triangle) and Stop signal (red triangle). The SSD changed depending on the participant’s accuracy on Stop trials. If a response was successfully inhibited, then inhibition was made more difficult on a subsequent Stop trial by increasing the SSD by 50 ms. If inhibition was unsuccessful, inhibition was made easier on the next Stop trial by decreasing the SSD by 50 ms. The SSD was determined using the tracking method where by four staircases were used to ensure that the probability of correct inhibition was approximately 50% at the end of the experiment. The four staircases were started with SSD values of 100, 150, 200, or 250 ms. An equal number of trials were presented at each of the four staircases and to the left/right of fixation. Each run consisted of 128 trials containing 96 Go trials and 32 Stop trials. Prior to scanning, participants completed one run of practice trials. Two runs of trials were then presented during fMRI scanning. SSD values were initialized to the final values from each previous run, including the run of practice trials. Go reaction was measured from the onset of the Go signal to the key press. The stop signal reaction time (SSRT) was calculated by subtracting the final SSD from the mean RT on Go trials. Higher SSRT values are indicative of slower inhibition. Higher SSD values indicate better inhibitory control. Both indices measure stopping speed or proficiency.

### Imaging Protocols

Imaging was conducted on a GE MR750 Discovery 3 Tesla system with an eight-channel head coil. Head motion was limited by foam pads inserted between the head and the coil. Visual stimuli were viewed through a NordicNeuroLab goggle system. Non-ferrous key pad devices interfaced with a computer recorded task performance during fMRI for off-line analysis.

High-resolution T1-weighted anatomical images maximized differentiation of the white and gray matter boundary (3D spoiled gradient-recalled at steady state, minimum full TE, 7.8 ms TR, 600 TI, 8° flip angle, 1-mm slices, 25.6 cm FOV). For fMRI, echo-planar images (EPI) were acquired in an oblique orientation (perpendicular to the anterior-posterior commissure) to minimize susceptibility artifacts, using a single-shot, blipped, gradient-echo, EPI pulse sequence (30.5 ms TE, 2.0 s TR, 90° flip angle, 25.6 cm FOV, 64 × 64 matrix, 37 contiguous 4 mm slices (3.75 × 3.75 × 4 mm voxel size) that provided coverage of the entire brain. Whole-brain axial diffusion tensor images (dMRI) were acquired on 3.0T GE MR750 using a single-shot EPI sequence with diffusion-encoding along 51 directions, *b-*value = 1000 s/mm2, six non-diffusion weighted images (*bo*), slice thickness 2.0 mm, TR = 9.2 s, TE = minimum, matrix = 128 × 128mm, FOV = 25.6 mms, and voxel size = 2.0 × 2.0 × 2.0 mm), and fat suppression.

### fMRI Analyses

#### Head Motion

Data were processed and analyzed using the Analysis of Functional NeuroImages (AFNI) software (Cox et al., 2017). After discarding the first 4 volumes of the time series, functional data were motion corrected using Slice-Oriented Motion Correction (SLOMOC) (Beall and Lowe, 2014), which performs an in-plane slice-wise motion registration, followed by an out-of-plane motion parameter estimation and regularization. Before motion correction, the groups did not differ in maximum scan-to-scan displacement (*F* = 2.7, *p* > 0.10, $\eta_{p}^{2}$ = 0.048; Control mean = 0.88 mm, *SD* = 0.46; PD mean = 0.69 mm, *SD* = 0.39), which was <2 mm in all subjects. Likewise, no group differences were found in framewise displacement (*F* < 1.0; $\eta_{p}^{2}$ = 0.01; Control mean = 0.23, *SD* = 0.11; PD mean = 0.25, *SD* = 0.11). Thus, procedures for restricting head motion were highly effective. Motion correction (SLOMOC) further reduced small fluctuations in head motion (i.e., <0.10 mm and <0.04 mm in all subjects for maximum and framewise displacement). After motion correction, the volumes were time shifted, transformed to Talairach space, and spatially filtered (6 mm Gaussian kernel).

#### Voxel-Wise Analyses of Condition Effects

First-level voxel-wise analyses tested for the effect of the task conditions on brain activation for each group to verify that during SST performance commonly reported regional patterns of brain activation were produced. We did not expect to find condition effects for the StN and SN, since whole-brain voxel-wise analyses are often insensitive to activation differences between conditions in small volumes. As such, the StN and SN were also included in our context-dependent connectivity analyses (see below). AFNI 3dDeconvolve was used to estimate the hemodynamic response function (HRF) of each voxel using multiple linear regressions. The analysis pipeline included deconvolution of each subject’s time series for each experimental condition [correct Go trials; correct or successful inhibitions (SI); and incorrect or failed inhibition (FI)] to generate an HRF of the signal on a voxelwise basis. Each HRF was estimated relative to the baseline state. Incorrect Go trials were regressed out of the time series at each voxel. The main dependent measures were the differences in magnitude of the signal (beta coefficient) for the SI > Go and the SI > FI conditions. The latter comparison controls for the greater salience of stop trials relative to Go trials and probes for activity related to stopping accuracy.

The effects of task condition on brain activation in each group (SI > Go; SI > FI) were tested using 3dMVM (Chen et al., 2014). Monte Carlo simulations with 5,000 iterations (3dClustSim using the ACF method) were conducted on a slightly inflated gray matter mask (14,209 4 mm × 4 mm × 4 mm voxels) to compute the voxel-probability and minimum cluster-size threshold needed to obtain a 0.05 familywise alpha (Cox et al., 2017). Because spatial thresholds are biased against small activation clusters in some regions of interest (ROI), thresholds were derived separately for caudate, SN, and StN volumes. A corrected alpha of *p* < 0.05 was obtained using a voxelwise probability of *p* < 0.002 and a minimum cluster size of ≥14.7 voxels for the cortex and ≥4.7 voxels for the basal ganglia.

#### Voxel-Wise Analyses of Group Differences in Activations for the Task Conditions

Based on the results from the first-level analyses, ROI maps were generated by combining the regions that showed a condition effect of interest for each group. Then second-level voxelwise ANCOVAs (sex adjusted) tested for group differences in regions that showed the above condition effects (3dMVM). Based on 5,000 Monte Carlo simulations (3dClustSim using the ACF method) of each condition map, a corrected alpha of *p* < 0.05 was obtained for tests of group differences using a voxelwise probability of *p* < 0.005 and minimum clusters sizes of 8 voxels (SI > Go) and 6.3 voxels (SI > FI).

#### Context-Dependent Connectivity Analyses (gPPI)

The main focus of the hypotheses was to test whether context-dependent connectivity of a seed ROI with other brain regions (i.e., SI versus Go; SI versus FI) differed between the groups. The generalized psychophysical interaction (gPPI) method, as implemented in AFNI software, was used since beta estimates are more sensitive and specific to context-dependent connectivity than the standard PPI method (McLaren et al., 2012; Cisler et al., 2014). 5 mm diameter seeds were constructed around the coordinates for each ROI, which were positioned so that they overlapped with regional activations that showed significant task condition effects in the first-level voxel-wise analyses of condition effects. The seeds and their coordinates for gPPI analyses included the (1) inferior frontal gyrus (BA 44: 53, 11, 12; BA 45: 54, 23, 10; BA 47: 47, 25, -10), (2) preSMA (0, -11, 50), (3) right caudate nucleus (9, 13, 0), (4) right StN (11, -13, -7), (5) right SN (SN: 8, -18, -5) (Aron, 2007), (6) the right anterior insula (34, 20, 4) (Hampshire and Sharp, 2015), and (7) a pivotal executive-control center involved in response inhibition (Jahanshahi et al., 2015b; Morein-Zamir and Robbins, 2015; Zhang et al., 2017), namely right DLPFC (BA 9). Individual time courses in the processed raw signal dataset were then extracted for each seed region and the hemodynamic delay was removed from the time courses (AFNI 3dTfitter). The resultant seed-region signal was multiplied by a condition of interest regressor, thereby creating an interaction time course, which was convolved with a gamma-variate HRF. The first regressor (physiological variable) represents the time series of activity from the seed ROI. The second regressor (psychological variable) represents the task condition (i.e., SI versus Go; SI versus FI). The PPI regressor is computed as the cross-product of the physiological and psychological variables. The regression model controlled for nuisance variables (baseline differences, linear drift, and 12 motion parameters), the task regressors, and the seed time-course. The regression produced correlation maps for the time course in the seed regions with the time course from all other brain voxels as a function of a condition of interest. Fisher *z* transforms were applied to the correlation maps to test for group differences in context-dependent connectivity. Group comparisons (adjusted for sex) were thresholded using a voxelwise-probability of *p* < 0.005 and minimum cluster size of 24 voxels for cortical connectivity and 8 voxels for subcortical connectivity (5,000 simulations using the ACF method). The false discovery rate (FDR) method was applied to the corrected p values to further adjust for analyses of multiple seeds.

### dMRI Analyses

Processing and analysis of dMRI data was conducted using Functional Magnetic Resonance Imaging of the Brain (FMRIB) software library 5.0.8 (FSL). After motion and eddy-current correction, data for each subject were fit on a voxel-by-voxel basis to the diffusion tensor model, accounting for floor bias with a maximum likelihood estimation approach. Fractional anisotropy (FA), mean diffusivity (MD), axial diffusivity (AD), radial diffusivity (RD) were calculated from the diffusion tensor in each voxel. Data were then processed using Tract-Based Spatial Statistics preprocessing functions (Smith et al., 2006). FA images were non-linearly registered to FMRIB58_FA for each subject and then to a study specific FA template in Montreal Neurological Institute atlas space. This process was repeated for MD, AD, and RD maps. ROI were created using the Johns Hopkins University (JHU)-International Consortium of Brain Mapping (ICBM) labels WM atlas, which contains 48 white-matter tracts that were hand-segmented on an average probabilistic tensor map of 81 healthy participants. Preliminary group by hemisphere ANCOVAs (sex adjusted) showed no group differences in dMRI metrics as a function of hemisphere. Thus, homologous hemispheric tracts were combined into a single bilateral ROI by multiplying diffusion metrics of a tract by each hemisphere volume, then summing the products and dividing by the total volume. Tracts of interest were identified for correlations with context-dependent connectivity variables. These analyses were constrained to tracts anatomically underlying context-specific functional connectivity patterns that differed between the groups (see Results).

### Volumetric MRI Analyses

Brain volumes were derived using the FreeSurfer 5.3 recon-all pipeline^1^. Briefly, each subject’s MRI volume was linearly registered to Talairach space, bias-field corrected and then a high-dimensional, non-linear registration to Talairach space was performed. Each voxel of the volume was automatically assigned a label based on probabilistic estimations relying on Markov random fields (Fischl et al., 2002). Group comparisons were conducted on bilateral cortical (frontal, parietal, occipital, and temporal) and basal ganglia volumes (caudate, putamen), since preliminarily analyses indicated that group differences did not vary between hemispheres. Volumetric measures were adjusted for total intracranial volume to account for individual differences in head size.

### Statistical Analyses

Owing to group differences in sex, analyses were conducted on sex-adjusted standardized residuals computed for behavioral (SST measures, neuropsychological tests) and MRI (rsfMRI and brain volume) variables. The FDR method adjusted for multiple analyses (*q* value < 0.05), except where noted. Post-processing of dMRI data regressed out sex effects, rendering it unnecessary to compute standardized residuals for these measures.

To determine if activation was associated with SST measures, regions comprising the right-hemisphere inhibition network that showed greater SI than Go activation were extracted for each subject. Functional and SST measures (SSRT; SSD) were converted to sex adjusted standardized residuals and then correlated, separately for each group. Pearson correlation analyses also tested for associations between SST measures and the standardized residuals of aberrant PPI measures that showed greater SI than Go connectivity. Owing to the *a priori* interest in neurobehavioral associations, analyses were not adjusted for multiple analyses. In the PD group, correlations were conducted to investigate relationships between SST variables (SSD, SSRT, PPI variables) and clinical variables (FDR adjusted).

Lastly, discriminant function analyses with classification were performed on MRI variables that significantly differed between the groups to identify sensitive signatures of neuropathological changes in PD, which could inform the development and refinement of measures that have potential to serve as markers in longitudinal studies. To reliably estimate classification accuracy, a bias-corrected accelerated bootstrap (1,000 bootstrapped samples) method was used (Efron, 1987). Receiver operating curve analyses (ROC) then evaluated the goodness-of-fit of the discriminant model in distinguishing PD from controls by analyzing the area under the curve (AUC) for the sensitivity and specificity distributions relative to the null hypothesis (AUC = 0.50).

## Results

### Neuropsychological Test Performance

Analyses of covariance (ANCOVA; sex adjusted) showed that the PD group had significantly lower scores than controls on the Letter Fluency test, but no other group differences were found on the remaining neuropsychological tests (Table 1). While the former finding indicates declining verbal fluency in PD at the group level, individual patients did not exhibit clinically significant cognitive decline indicative of MCI. Self-reports of daytime sleepiness (Epworth Sleepiness Scale) and depression symptoms (Hamilton Depression Scale) did not differ between the groups. Depression symptoms in both groups were within the normal to mild range (0–8).

### Stop Signal Task Performance

ANCOVA (adjusting for sex) was used to test for group differences in the SST measures (Table 2). Stop trials were approximately evenly divided between correct and incorrect inhibitions, indicating that the four staircases were effective in producing about 50% correct inhibitions. The percent correct stops (inhibitions) did not significantly differ between the PD and control groups. Most subjects showed between 40 and 60% correct inhibitions, except two PD participants with higher percentages (66 and 67%) that were still acceptable for obtaining reliable estimates of SSRT (Congdon et al., 2012). In both groups, performance on Go trials was highly accurate (≥95%), and omission errors on Go trials did not differ between groups. Go RT also did not differ between the groups. Outliers were not found in Go RT distributions of either group, indicating that distributions were not unduly skewed. Ancillary analyses demonstrated that in both groups, mean RT on Go trials was significantly longer than on failed stop trials (Controls: *F* = 90.1, *p* = 2.99E-10; PD: *F* = 112.8, *p* = 3.84E-11), consistent with the independence assumption of the race model of stopping (Logan et al., 1984). This difference was similar between the groups (group × trial type interaction: *F* = 2.8, *p* = 0.10). Moreover, all participants showed longer Go RTs than failed mean RTs. There were no significant group differences in the main measures, namely SSRT and SSD. SSRT did not correlate with Go RT in either group (Control: *r* = -0.23, *p* = 0.22; PD: *r* = -0.27, *p* = 0.15), indicating that slowing did not significantly affect SSRT estimates. Altogether, these results are consistent with the independence assumption of the race model, which generally provides a good account of stop-signal performance in a variety of tasks and different populations (Verbruggen and Logan, 2009; Congdon et al., 2012; Jilka et al., 2014; Wessel et al., 2016; Bartoli et al., 2018).

**Table 2:** Stop signal task performance.

	Parkinson’s	Controls	*p*	$\eta_{p}^{2}$
Go percent correct	95.0 (7.0)	97.5 (4.1)	0.10	0.04
Go omission errors	4.5 (6.9)	2.1 (4.0)	0.11	0.04
Go correct RT (ms)	599.8 (133.8)	543.2 (135.7)	0.13	0.04
Failed Stop RT (ms)	530.9 (129.7)	488.9 (119.4)	0.21	0.03
Stop percent correct	54.6 (6.7)	51.2 (8.6)	0.09	0.05
SSRT (ms)	252.1 (48.4)	242.5 (30.8)	0.38	0.01
SSD	347.7 (155.7)	300.6 (145.9)	0.24	0.03


*Tabled values are group means and standard deviations. Group differences on each variable were tested using ANCOVA (sex adjusted). SSD, stop signal delay; SSRT, stop signal reaction time estimated using the mean method (Verbruggen and Logan, 2009).*

### fMRI Data

#### Voxelwise Tests of Condition Effects

**FIGURE 1:**
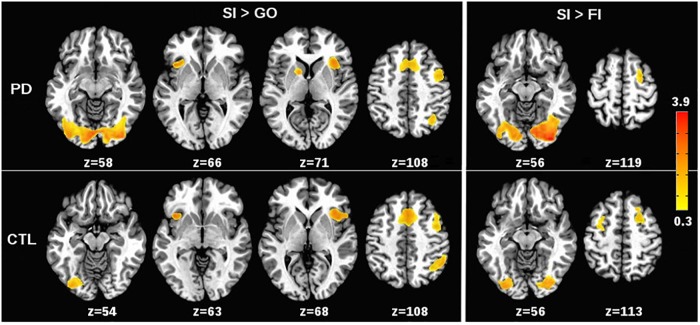
Effects of stop signal task condition on voxelwise tests of brain activation. **(Left)** (SI > Go) shows regional activations that were greater for correct or successful inhibition (SI) than for Go trials in the PD and control (CTL) groups. **(Right)** (SI > FI) shows regional activations that were greater for SI than for incorrect or failed inhibitions (FI) in the PD and CTL groups. The significant effects of task condition on brain activation (*p* < 0.002) were generally similar for the PD and control groups. The color bar displays the range of beta values for significant condition effects.

Patterns of condition effects (*p* < 0.002) were typically similar for the PD and control groups (Figure 1). In both groups, activation was greater for SI than Go trials within the response inhibition network including the preSMA and right IFG (BA 44, 45, 47), and the anterior insula (bilateral). In both groups, SI > Go activation was observed in the left IFG (BA 44, 45, 47), right middle frontal and DLPFC (BA 6, BA 9), right inferior parietal cortex, and bilateral lingual gyrus. Only the PD group showed SI > Go activation in the left caudate. As for the SI > FI activation, inhibition failures in both groups were typified by deactivation of the bilateral lingual gyrus and right middle frontal gyrus (BA 6). In the control group, inhibition failures were also associated with reduced activation of the left middle frontal gyrus (BA 6).

To determine if brain activity during successful inhibitions relative to Go trials was associated with inhibition proficiency, individual variations in activation of the inhibition network (preSMA, right BA 44, 45, 47, right anterior insula) were correlated with SSD and SSRT, separately for each group. In the control group, greater SI than Go activation in right BA 44 was associated with lower SSD values (worse inhibitory control) (*r* = -0.37, *p* = 0.05). No other correlations were significant. In the PD group, greater SI activation in the preSMA and the right BA 44 were associated with lower SSD values (*r* = -0.39, *p* = 0.04 and *r* = -0.38, *p* = 0.05, respectively) and prolonged SSRT (worse) (*r* = 0.47, *p* = 0.01 and *r* = 0.43, *p* = 0.026, respectively). In addition, greater SI than Go activation of the right anterior insula was associated with lower SSD values (*r* = -0.39, *p* = 0.04). Response inhibition performance was not associated with SI > FI activation in either group.

#### Voxelwise Tests of Group Differences

Relative to controls, the PD group showed greater activation in the right lingual gyrus for SI trials in comparison to Go (*p* < 0.0001) and FI trials (*p* < 0.0006). No other significant group effects were found.

#### Context-Dependent Connectivity (gPPI)

**FIGURE 2:**
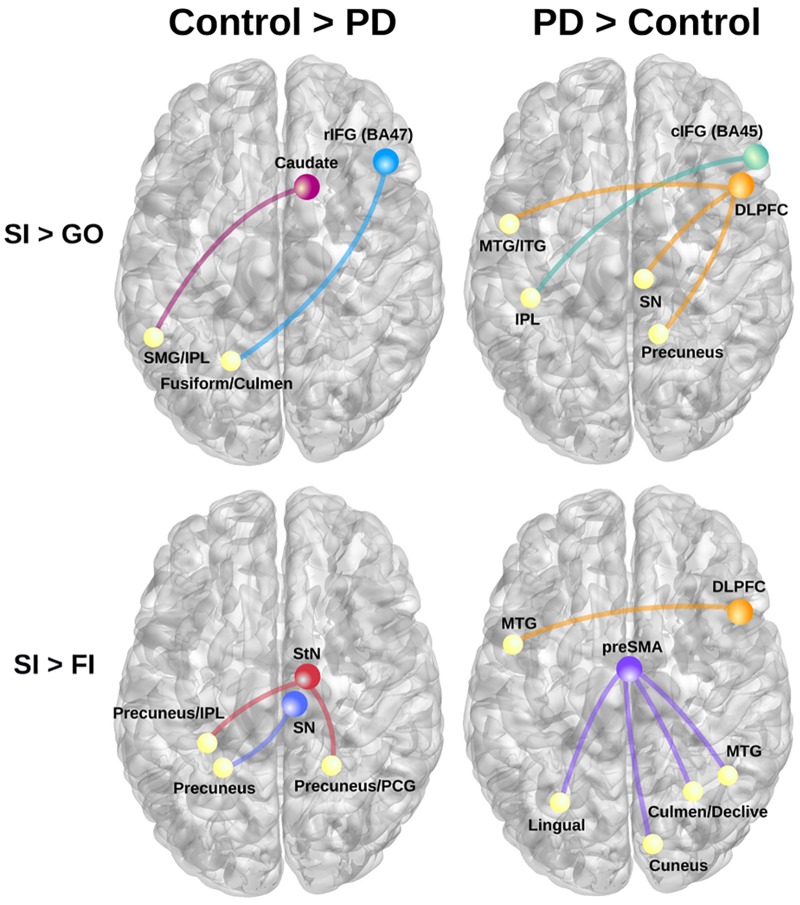
Group differences in context-dependent connectivity as modulated by successful inhibitions (SI) relative to Go trials and failed inhibition (FI) trials. **(Left)** Connectivity patterns that were stronger in the control group for SI than Go trials (**top row**; SI > Go) and SI than FI trials (**bottom row**; SI > FI). **(Right)** Displays connectivity patterns that were stronger in the PD group for SI than Go trials (**top row**; SI > Go) and SI than FI trials (**bottom row**; SI > FI). Colored circles/lines show seed regions of interest and their connections are designated by yellow circles. BA, Brodmann area; cIFG, caudal inferior frontal gyrus (BA 45); DLPFC, dorsolateral prefrontal cortex (BA 9); IPL, inferior parietal; ITG, inferior temporal gyrus; MTG, middle temporal gyrus; PCG, posterior cingulate gyrus; rIFG, rostral inferior frontal gyrus (BA 47); preSMA, pre-supplementary motor area; SMG, supramarginal gyrus; SN, substantia nigra; StN, subthalamic nucleus.

Table 3 details the significant group differences in context-dependent connectivity as it was modulated by successful inhibitions relative to Go and FI trials. Effect sizes for all tests of significant group differences were very large ($\eta_{p}^{2}$ ≥ 0.18). Figure 2 (top row) shows that in the control, but not PD group, successful inhibitions were distinguished from Go trials by stronger connectivity of the right rostral IFG (rIFG; BA 47) and right caudate with visual areas (fusiform gyrus), the cerebellum, and the dorsal attention network (inferior parietal, IPL). In contrast, right caudal IFG (cIFG; BA 45) and DLPFC connectivity with dorsal attention (IPL), ventral attention (temporal), the default mode network (DMN; precuneus), and a subcortical inhibition center (SN) was stronger for SI than Go trials in the PD, but not the control group. Figure 2 (bottom row) shows group differences in context-dependent connectivity that depended on stopping accuracy (SI > FI). In the control group, successful stops were distinguished from failed stops by stronger connectivity of the StN and SN largely with the DMN [precuneus, posterior cingulate gyrus (PCG)]. In the PD group, successful stops differed from failed stops by stronger connectivity of DLPFC and preSMA with ventral attention systems (temporal-occipital) and the cerebellum. No other seeds showed group differences in context-dependent connectivity.

**Table 3:** Group differences in context-dependent connectivity as modulated by successful inhibitions relative to Go and failed inhibition trials.

Seed	Region	Voxels	*X*	*Y*	*Z*	*p*-value	$\eta_{p}^{2}$
**SI > Go in Controls**							
R rIFG (BA 47)	L fusiform and culmen	37	-20	-57	-10	0.0001	0.25
R caudate	L SMG and IPL	28	-50	-49	25	0.0001	0.24
**SI > Go in PD**							
R cIFG (BA 45)	L IPL	33	-40	-35	50	0.00004	0.27
R DLPFC	L MTG and ITG	24	-47	-2	-27	8.20E-8	0.46
	R precuneus	23	13	-50	63	0.0003	0.22
	R SN	12	8	-23	-14	0.001	0.18
**SI > FI in Controls**							
R SN	L precuneus	64	-20	-49	29	0.00001	0.32
R StN	L precuneus and IPL	332	-26	-38	28	0.000002	0.35
	R precuneus and PCG	267	24	-46	27	0.000003	0.34
**SI > FI in PD**							
R DLPFC	L MTG	25	-45	1	-27	8.28 E-7	0.37
preSMA	R cuneus	131	11	-78	7	2.14 E-7	0.40
	R MTG	107	42	-52	0	6.78 E-7	0.37
	L lingual gyrus	59	-25	-64	7	4.11 E-7	0.38
	R culmen and declive	47	27	-55	-16	0.00002	0.29


**X, Y, Z* coordinates are based on the Talairach atlas. *P* and η_*p*_^*2*^ values are from tests of group differences. BA, Brodmann area; cIFG, caudal inferior frontal gyrus (BA 45); DLPFC, dorsal lateral prefrontal cortex (BA 9); FI, failed inhibition trials; L and R, left and right hemisphere; IPL, inferior parietal lobule; ITG, inferior temporal gyrus; MTG, middle temporal gyrus; PCG, posterior cingulate gyrus; rIFG, rostral inferior frontal gyrus (BA 47); SI, successful inhibition trials; SMG, supramarginal gyrus; SN, substantia nigra; StN, subthalamic nucleus.*

Next, we examined relationships (sex adjusted) between context-dependent connectivity and the speed or proficiency of inhibition, as measured by SSRT and SSD. In the control group, stronger right caudate – left IPL connectivity for SI trials (relative to Go) was associated with slower SSRTs (worse) (*r* = 0.45, *p* < 0.015); no such association was found in the PD group (*p* > 0.29). In the PD group, stronger rIFG – left fusiform/culmen connectivity was associated with higher SSDs (better) (*r* = 0.46, *p* < 0.015); no such relationship was found in the control group (*p* > 0.26). As for SI > FI connectivity, stronger right SN and StN connectivity with left precuneus/IPL for SI trials was associated with slower SSRTs in the control group (*r* = 0.43, *p* < 0.02 and *r* = 0.39, *p* < 0.40, respectively). No significant relationships between inhibition proficiency measures and SI versus FI connectivity were found in the PD group (*p* > 0.15).

### dMRI Data

Group differences in diffusion metrics (FA, MD, AD, RD) were not found for any white-matter tracts. We then examined if individual variations in tissue diffusivity within each group correlated with measures of aberrant context-dependent connectivity. We focused on aberrant PPI variables associated with greater SI than Go connectivity (Table 3; 6 PPI variables). FA was used for these analyses as it is a stable metric that measures the normalized variance of the three diffusion scalars. Lower FA values in PD typically signify reduced white-matter integrity (e.g., axonal degeneration and/or demyelination) (Theilmann et al., 2013).

#### Abnormal Context-Dependent Connectivity and Diffusivity in Underlying Tracts

Correlations were constrained to analyses of FA in tracts underlying the group differences in corticostriatal and cortico-cortical context-dependent connectivity for successful inhibitions relative to Go trials. The analyses included the following tracts: (1) the corticospinal tract (CST), which is concerned with motor function; (2) the anterior and posterior limb of the internal capsule (ALIC; PLIC) and external capsule (EC), projection tracts containing connections from the thalamus/basal ganglia to the cerebral cortex; (3) the superior longitudinal fasciculus (SLF), a major association tract that supports cortico-cortical communication; and (4) the body of corpus callosum (CC), a commissural tract that supports interhemispheric interactions. Pearson correlations were conducted separately for each group and FDR adjusted for multiple analyses over six tracts for each PPI variable.

**FIGURE 3:**
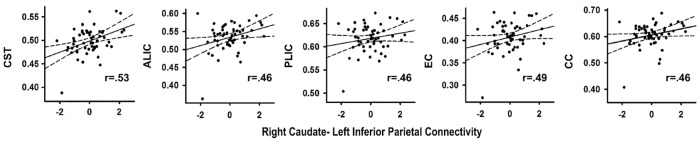
Association between abnormal context-dependent connectivity of the right caudate - left inferior parietal cortex and fractional anisotropy (FA) in underlying tracks in the PD group. The *x* axis plots the standardized residuals (adjusted for sex) for right caudate-left inferior parietal cortex connectivity. The y axis plots FA values (adjusted for sex during data post-processing) in bilateral white matter tracks. Solid lines show the best-fitting linear regression line and dashed lines represent the 95% confidence intervals. Pearson correlation coefficients are displayed in the lower right corner of each scatter plot. ALIC, anterior limb of the internal capsule; CC, body of the corpus callosum; CST, corticospinal tract; EC, external capsule; PLIC, posterior limb internal capsule.

In the PD group, stronger right caudate connectivity with the left inferior parietal cortex was associated greater FA in the CST (*r* = 0.53, *p* < 0.004), ALIC (*r* = 0.46, *p* < 0.015), PLIC (*r* = 0.46, *p* < 0.017)), EC (*r* = 0.49, *p* < 0.010) and CC (*r* = 0.46, *p* < 0.017) (Figure 3). No such associations were found in the control group (*p* > 0.20). Significant associations were not found between the other PPI variables and FA for either group. In the PD group, FA in tracts did not correlate with SSD or SSRT. In controls, higher FA in the ALIC (*r* = -0.48, *p* < 0.01) and the CC (*r* = -0.59, *p* < 0.001) was associated with faster response inhibition (SSRT).

### Brain Volumes

Frontal lobe volume was significantly smaller in the PD than the control group (*F* = 9.2, *p* < 0.005, $\eta_{p}^{2}$ = 0.14), but no group differences were found for parietal, occipital, temporal, or basal ganglia (caudate, putamen) volumes (FDR adjusted).

#### Abnormal Context-Dependent Connectivity and Brain Volumes

**FIGURE 4:**
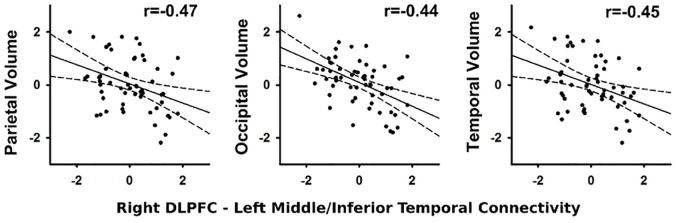
Association between abnormal context-dependent connectivity of the right DLPFC (BA 9) – left middle/inferior temporal gyrus and brain volumes. The *x* and *y* axes plot the standardized residuals (adjusted for sex) for context-dependent connectivity and brain volumes. Solid lines show the best-fitting linear regression line and dashed lines represent the 95% confidence intervals. Pearson correlation coefficients are displayed in the upper right corner of each scatter plot.

In the PD group, stronger connectivity of the right DLPFC with left middle/inferior temporal cortex during successful inhibitions (relative to Go trials) was associated with reduced parietal (*r* = -0.47, *p* < 0.012), temporal (*r* = -0.45, *p* < 0.018), and occipital (*r* = -0.44, *p* < 0.019) volume, but not frontal lobe or basal ganglia volume (Figure 4). No such associations were found in the control group (*q* value > 0.05). Associations were not found between the other PPI variables and regional volumes for either group (FDR adjusted). In both groups, larger temporal (Control: *r* = -0.44, *p* < 0.016; PD: *r* = -0.44, *p* < 0.019) and parietal (Control *r* = -0.43, *p* < 0.02; PD: *r* = -0.47, *p* < 0.01) lobe volumes were associated with faster response inhibition (SSRT).

### Associations Between Cognitive, Clinical, and PPI Variables

In the PD, but not control group, slower SSRTs correlated with poorer executive functioning, including worse inhibition and cognitive flexibility (DKEFS Color-Word Interference/Switching; *r* = 0.62, *p* < 0.0004), attention (Adaptive Digit Ordering; *r* = -0.41, *p* < 0.03), and verbal fluency (*r* = -0.38, *p* < 0.045) (FDR adjusted). No relationships were found with SSD. Disease duration and levodopa dosage equivalence did not significantly correlate with SST measures, nor did motor symptoms (UPDRS total motor, tremor, PIGD) ON or OFF medication. PPI measures did not correlate with neuropsychological or clinical variables.

### Sensitivity of MRI Variables

Table 4 summarizes the discriminant and ROC analyses that were performed on the two sets of PPI variables [Table 3: SI > Go (6 variables); SI > FI (8 variables)] and frontal lobe volume, which was reduced in the PD group. Context-dependent connectivity markers of group differences exhibited excellent overall accuracy (AUC ≥ 0.96) in distinguishing a PD patient from healthy individuals. Frontal lobe volume showed poor accuracy (AUC = 0.72).

**Table 4:** Sensitivity, specificity, and accuracy of classification.

	% Correct Classification		Discriminant Function Centroid^a^		Chi-square^b^	AUC (CI)^c^
				
Variable Set	Control	PD	Control	PD		
**Context-dependent connectivity**						
SI > Go (6 variables)^d^	90	89	-1.21	1.25	49.1	0.96 (0.91 – 1.00)
SI > FI (8 variables)^d^	83	96	-1.22	1.26	48.6	0.97 (0.94 – 1.00)
**Brain volume**						
Frontal lobe volume	66	64	0.42	-0.43	9.3	0.72 (0.59 – 0.85)


*^*a*^The centroid is the mean of the discriminant function score for each group. ^*b*^Chi-square tests of group differences were significant for discriminant function analyses of each set of task-activated fMRI PPI variables (*p* ≤ 7.4E-8) and for frontal lobe volume (*p* = 0.002). ^*c*^AUC tests indicate that average accuracy of classification was significantly greater than random for each tafMRI variable set (*p* < 2.9E-9) and for frontal lobe volume (*p* = 0.005). CI = 95% confidence interval. ^*d*^Table 3 lists the specific context-dependent connectivity variables that showed significant group differences in the gPPI analyses for SI > Go and SI > FI. FI, failed inhibition trials; SI, successful inhibition trials.*

## Discussion

We found for the first time that context-dependent connectivity of inhibition centers differed between the groups, such that functional reorganization in PD was related to aberrant long-range or between-network connections with temporal, parietal, and occipital networks, rather than altered interactions within the inhibition network. Successful inhibition in PD was uniquely characterized by strengthened context-dependent connectivity of cortical inhibition centers (DLPFC, cIFG, and preSMA), whereas successful inhibition in controls was distinguished by strengthened context-dependent connectivity of the rIFG and basal ganglia (caudate, SN, and StN). In controls, stronger connectivity of the basal ganglia, but not the rIFG, was related to inhibition proficiency and/or accuracy (accurate versus failed stopping). By comparison, in PD aberrantly stronger DLPFC and preSMA connectivity was associated with inhibition accuracy, but not proficiency. However, patients who exhibited more proficient inhibitory control also showed a pattern of stronger rIFG connectivity that was found in the control group. The absence of a relationship between stronger connectivity and inhibition proficiency, but not accuracy, may relate to reduced coherence in functionally reorganized systems, compensatory mechanisms, and/or the reorganization of intrinsic networks. In PD participants only, poorer executive functioning also correlated with worse response inhibition, but not aberrant context-dependent connectivity. This too indicates that contextually dependent functional reorganization does not necessarily correlate with all facets of cognitive control. Connectivity measures were highly sensitive in distinguishing PD from control participants, unlike the magnitude of fMRI activation within the inhibition network (voxel-based fMRI) and tissue diffusivity (FA), which did not differ between the groups, and frontal lobe volume, which showed poor classification accuracy. Lastly, the strength of some effective connectivity measures during successful inhibition was partly related to structural variations in the brain of PD participants, but not controls, despite an absence of group differences in these measures. This result may suggest that altered connectivity of the inhibition network presages future changes in underlying brain structure.

### Aberrant Context-Dependent Connectivity

Recently, dynamic causal modeling (DCM) among four inhibition regions (preSMA, IFG, StN, motor cortex) failed to characterize the interactions amongst frontal and subcortical inhibition areas in PD ON participants (Rae et al., 2016), as it did in the control group. This negative result was thought to be due to the heterogeneity amongst patients (Rae et al., 2016). Our results suggest that DCM may not have successfully modeled connectivity in PD, partly due to the omission of the DLPFC and SN, which support inhibitory control (Jahanshahi et al., 2015b; Morein-Zamir and Robbins, 2015; Zhang et al., 2017). In this regard, we found that DLPFC connectivity with the right SN was strengthened in PD for successful inhibitions. Modeling network interactions amongst all key regions is important, especially in clinical disorders, since inhibitory control is comprised of multiple sub-processes (e.g., attention, conflict resolution, response preparation, action cancelation) (Sebastian et al., 2013) that may be selectively altered in PD and affect within-network interactions. PD heterogeneity could also be reduced by screening for MCI, for which impaired performance and weakened functional connectivity is more characteristic of advancing disease progression (Olde Dubbelink et al., 2014). Indeed, impaired response inhibition and weakened right IFG- striatal functional connectivity was found in PD patients who were not screened for MCI (Ye et al., 2015), which contrasts with our findings of normal response inhibition and preserved within inhibition network connectivity. At the same time, findings from the gPPI method cannot be directly compared to those obtained from DCM, which allows for hypothesis-driven tests between different models of cause-effect interactions within a network comprised of multiple brain regions. Thus, the failure of DCM to characterize network interactions in PD may partly relate to large individual differences in cause-effect interactions within a defined brain network (Rae et al., 2016), which cannot be modeled using the gPPI method. Nonetheless, leveraging both approaches may promote a better understanding of the functional architecture of the inhibition network, especially in disease states where reorganization of function can involve interactions amongst regions that are not found in healthy participants (Harrington et al., 2017), and better characterize the nature of regional interactions within large-scale networks and their modulation by behavioral contexts (Limongi and Perez, 2017).

To the best of our knowledge this is the first study of PD to demonstrate a context-dependent functional reorganization in the long-range connectivity of regions that are frequently implicated in response inhibition processes. Aberrantly strengthened connectivity was largely with temporal-occipital and parietal cortices, which are elements of the ‘bottom–up’ ventral and ‘top–down’ dorsal attention networks, respectively (Corbetta and Shulman, 2002; Wu et al., 2015). Specifically, connectivity of the right cIFG, which governs executive processes including action updating and attention switching (Hampshire, 2015), was aberrantly strengthened for successful inhibitions (relative to Go trials) with the dorsal ‘controlled attention’ network (left IPL). In contrast, DLPFC connectivity with the middle/inferior temporal gyrus was abnormally strengthened in PD for successful inhibitions relative to both Go trials and failed stopping. Similarly, connectivity of the preSMA, which mediates action planning (Elsinger et al., 2006), was aberrantly stronger with temporal-occipital areas when stopping was successful than when it failed. Thus, key elements of the inhibition network showed strengthened interactions with controlled- and stimulus-driven attention networks, likely because stop trials require a switch from relatively automatic (Go trials) to highly controlled behavior. At the same time, we also found that DLPFC connectivity was strengthened in PD with the right precuneus, a region of the DMN, for which stronger activation in healthy adults is associated with slower inhibitory control (Congdon et al., 2010) and more generally, lapses in focused attention (Whitfield-Gabrieli and Ford, 2012). Frontal interactions with the DMN may adversely affect inhibitory control as the disease progresses and attentional control declines. This speculation is compatible with reports of abnormally enhanced positive coupling between the DMN and the salience and central executive networks in PD (Putcha et al., 2015, 2016), especially in patients who are less adept at switching attention (Putcha et al., 2016).

Interestingly, inhibition proficiency in PD was not correlated with abnormally stronger connectivity of cortical inhibitory control centers. One explanation is that some context-specific changes (i.e., successful inhibitions versus Go trials) are fundamentally due to intrinsic network reorganization in PD, which can influence cognitive control (Whitfield-Gabrieli and Ford, 2012). To evaluate this prospect, associations between intrinsic and task-based connectivity should be investigated. However, if this was a main factor underlying context-specific reorganization then connectivity might be expected to correlate with indices of disease severity (i.e., duration, levodopa dosage equivalence, motor symptoms), which was not found. In this regard, intrinsic and task-evoked connectivity are not simply two manifestations of the same underlying neuronal phenomenon (Gonzalez-Castillo and Bandettini, 2018). For example, task performance routinely produces stronger connectivity amongst networks that are recruited by a task and are otherwise disconnected in intrinsic networks (Cole et al., 2014; Shine et al., 2016). Moreover, we found that abnormally stronger DLPFC and SMA connectivity correlated with inhibition accuracy. Thus, the absence of a relationship between connectivity and inhibition proficiency, but not accuracy, may be accounted for by other mechanisms. One possibility is that aberrantly strengthened context-specific connectivity signifies difficulties in modulating interactions of inhibition nodes during effortful cognition, owing to reduced fidelity or coherence of connectivity within functionally reorganized systems. Aberrantly strengthened connectivity may also help sustain cognitive functions, postponing the onset of cognitive decline (Reuter-Lorenz and Park, 2014). Such mechanisms might not necessarily correlate with individual differences in the proficiency by which a task is accurately performed, although this should be examined using a longitudinal study design.

As for healthy adults, dorsal and ventral attention networks are activated by different inhibition processes (e.g., action selection, interference resolution, action cancelation, action withholding), although to different degrees (Sebastian et al., 2013; Zhang et al., 2017), indicating that inhibitory control is dynamic and influenced by multiple processes (Wiecki and Frank, 2013). In our controls, strengthened connectivity of inhibition centers (rIFG; subcortical areas) was especially notable with parietal cortex, but also higher-order visual processing and motor centers (fusiform, culmen). Interestingly, PD participants who were more proficient at stopping a response (longer SSD) also showed stronger rIFG-fusiform/culmen connectivity. This finding suggests that enhanced IFG connectivity with visual/motor areas can facilitate action cancelation in PD, possibly owing to a faster accumulation of evidence from the stop signal, which should lower the threshold for canceling a response (Wiecki and Frank, 2013).

With respect to the above findings, it is noteworthy that interventional treatments for deficient inhibitory control that target executive processes have only a modest effect on behavior (Marteau et al., 2012). Yet interventions that largely bypass executive processes by targeting automatic associative-process to behaviorally relevant stimuli, which are supported by the ventral attention network (De Pretto et al., 2017), appear more potent because they shape the development of highly routine inhibition-triggering behaviors to environmental stimuli (Houben, 2011; Houben and Jansen, 2011; Veling et al., 2011). Thus, strengthened connectivity of the inhibition nodes with the ventral attention network may help maintain inhibitory control in PD.

We also found that strengthened caudate, SN, and StN connectivity with the dorsal attention and DMN was stronger for inhibition successes than failures in controls, but not in PD patients. The StN is the subcortical node of the fast hyperdirect pathway that receives direct input from the prefrontal cortex, allowing cortical input to directly influence the StN (Wiecki and Frank, 2013; Jahanshahi et al., 2015a), which inhibits thalamocortical drive (Wessel et al., 2016) and in doing so, is the quickest route for stopping an action or pausing to accumulate more evidence for the correct course of action. Optogenetic stimulation of the StN demonstrates a causal role for this area in delaying or overriding prepotent behaviors (Fife et al., 2017). Importantly, in our control group stronger connectivity of both the StN and SN with the DMN (bilateral precuneus/PCG) distinguished inhibition successes from failures and was stronger for participants who had more difficulty successfully canceling a response (slower SSRTs). These results are consistent with the association between slower SSRTs and greater DMN activity in healthy adults (Congdon et al., 2010). Similarly, stronger right caudate-dorsal attention network (left SMG/IPL) connectivity was also associated with slower stopping ability in the control group. Altogether, the above findings suggest that failure to disengage the influences of controlled- or task-irrelevant (DMN) attention on the hyperdirect pathway may raise the decision threshold to prevent prepotent responding (Wiecki and Frank, 2013). In contrast, the hyperdirect pathway and cortical interactions with the caudate and SN did not modulate inhibition successes in PD participants. These findings may relate to testing patients OFF their medications, which could hamper the ability of basal ganglia nuclei to recruit and/or effectively communicate with cortical networks. A caveat is that successful inhibition in PD was related to aberrantly strengthened DLPFC connectivity with the right SN, suggesting that successful inhibitory control was partly mediated by top–down facilitation of dopaminergic neurons (Wiecki and Frank, 2013), which modulate detection of relevant cortical activity (Merchant et al., 2013).

Surprisingly, group differences were not found in the connectivity strength of the right anterior insula, a key region of the salience and inhibition networks. Although the magnitude of right anterior insula activation also did not differ between the groups, greater insula activation during successful inhibitions (relative to Go trials) was associated with worse inhibition proficiency (lower SSD) in PD participants, but not controls. We speculate that this disease-specific neurobehavioral association in cognitively normal PD may presage the future development of response inhibition deficits. This observation is important as the level of striatal dopamine depletion in PD-MCI is linked to D2 receptor availability in the insula, which in turn correlates strongly with poorer executive functioning (Christopher et al., 2014). Longitudinal studies are needed to sort out the meaning of neurobehavioral associations in relationship to disease progression.

### Association Between Aberrant Connectivity and Underlying Brain Structure

We reported earlier (Theilmann et al., 2013) that tissue diffusivity was abnormal in PD throughout anterior and posterior tracts, but patients were not screened for MCI (Litvan et al., 2012). Several recent studies of PD without MCI report normal tissue diffusivity (Hattori et al., 2012; Chen et al., 2015; Duncan et al., 2016). Despite an absence of group differences in tissue diffusivity in the present study, patients who showed stronger right caudate-left parietal connectivity during successful inhibitions, exhibited greater FA in tracts connecting the thalamus/basal ganglia to the cerebral cortex (ALIC, PLIC, EC) and supporting motor control (CST) and interhemispheric interactions (CC). Thus, structural disconnection within these tracts may undermine the enhancement of right caudate-left parietal connectivity, which distinguished successful inhibition in control participants and was associated with slower response cancelation (slower SSRT). Contrary to studies of non-demented PD participants (Ye et al., 2015; Rae et al., 2016), we found no association between FA and context-specific connectivity of the right IFG, likely because the PD sample in these earlier studies evidenced significant response inhibition deficits and white-matter abnormalities.

As for group differences in brain volume, only frontal lobe volume was significantly reduced in the PD group, contrary to other studies of PD without MCI (Tessitore et al., 2012; Baggio et al., 2015; Pirogovsky-Turk et al., 2015; Harrington et al., 2017). Despite this finding, smaller parietal, temporal, and occipital volumes were associated with stronger right DLPFC-left middle/inferior temporal connectivity only in PD participants. In both groups, however, smaller temporal and parietal volumes were also related to greater difficulty in canceling an ongoing response (slower SSRT), possibly suggesting that subtle reductions in volume render top–down and bottom–up attentional processing more unstable, which hampers rapid switches to unexpected behavioral goals. Although PD-related structural variations in white- and gray-matter tissue were associated with different facets of connectivity, this likely reflects the more direct structural-functional overlap between FA in subcortical projection and commissural tracts that underlie corticostriatal connectivity, and posterior cortical volumes that are related to frontal-temporal connectivity. Still, it was surprising that individual differences in FA of the SLF, which supports cortico-cortical communication, were not related to aberrant cortical-cortical connectivity in PD.

### Limitations

Our findings may be partly related to testing patients OFF medication, which produces greater disturbances in brain activation during response inhibition than when patients are tested ON medication (Farid et al., 2009). However, despite the lingering effects of dopamine after short-term medication withdrawal, levodopa dosage equivalence was not correlated withMRI or behavioral variables, suggesting this factor may not have had a large effect on the results. Since the effect of dopamine on response inhibition performance in PD remains debated (Obeso et al., 2011b; Manza et al., 2017), drug naïve patients would be a more ideal group to study.

Another limitation is that participants attained a higher educational level than is typical, so that the results may not generalize to all PD patients. Our recruitment methods were not biased toward highly educated individuals, as we recruited individuals throughout the San Diego area (e.g., Veterans, Parkinson’s support groups, community organizations). It is possible that greater cognitive reserve helps people to better cope with brain pathology by sustaining normal performance levels on the inhibition task (Muslimovic et al., 2009; Hindle et al., 2014; Lucero et al., 2015). To fully address this issue, future studies should assess the interactions of proxies for cognitive reserve (e.g., years of education, occupation, premorbid intelligence) on both behavior and brain structure and function.

## Summary and Conclusion

Our results demonstrate that functional reorganization of inhibition regions in PD precedes the development of inhibition deficits and clinically significant cognitive impairment. Functional reorganization was typified by abnormally enhanced long-range connectivity rather than altered within-network connectivity, which was preserved. Successful inhibition was related to strengthened connectivity of executive (DLPFC, cIFG) and action planning (preSMA) centers, largely with attention-related networks that have been the focus of some behavioral interventions for deficient inhibitory control. Amplified interactions of inhibition regions with attention networks may help sustain inhibitory control by postponing the onset of cognitive decline, although other explanations are possible. Unlike controls, PD participants also did not make use of the hyperdirect pathway or basal ganglia, to stop ongoing actions, possibly owing to dopaminergic cell loss. As disease severity progresses, one might expect that difficulties engaging these key subcortical pathways would lead to the development of response inhibition deficits. Interestingly, despite no group differences in tissue diffusivity or posterior cortical volume, individual differences in these structural variables correlated in a meaningful manner with some aberrant functional interactions in PD. These findings suggested that subtle decreases in underlying tissue diffusivity or brain volume may, respectively, undermine the enhancement of normal cortical-striatal connectivity or cause a strengthening in cortical-cortical connectivity. Lastly, context-dependent connectivity measures were highly accurate in distinguishing patients from controls, suggesting that they may be early markers of neuronal changes. Longitudinal studies are needed to directly test the above proposals.

## Author Contributions

DLH designed the study, conducted statistical analyses, interpreted the data, and assumed the lead role in writing the paper. She is the principal investigator of the primary Department of Veterans Affairs Merit Award. QS analyzed the task-activated fMRI data, consulted on the anatomical MRI analyses, and reviewed the manuscript. RJT analyzed the diffusion tensor imaging data and reviewed the manuscript. GNC contributed to the project design, collected the data, consulted on the analyses of task-activated fMRI and anatomical MRI data, and reviewed the manuscript. IL contributed to conceptual discussions about the study results and edited important intellectual content of the paper. JVF contributed to data collection, conceptual discussions about the study results, and edited important intellectual content of the paper. MH consulted on the diffusion tensor imaging analyses and edited important intellectual content of the paper. RRL consulted on the anatomical MRI analyses and edited important intellectual content of the paper.

## Conflict of Interest Statement

The authors declare that the research was conducted in the absence of any commercial or financial relationships that could be construed as a potential conflict of interest.

## Footnotes

1 http://surfer.nmr.mgh.harvard.edu/
